# Social Support Network, Neighborhood Social Cohesion, and Psychological Distress Among Caregiving Spouses

**DOI:** 10.1093/geroni/igaf122.3325

**Published:** 2025-12-31

**Authors:** Yeon Jin Choi, Seungjong Cho, Jihyeong Jeong

**Affiliations:** University of Kentucky, Lexington, Kentucky, United States; Texas Tech University, Lubbock, Texas, United States; University of Maryland Baltimore, Baltimore, Maryland, United States

## Abstract

Caregiving burden and stress are well-documented risk factors for poor mental health among caregiving spouses. While social support from family and friends can help alleviate caregiver distress, those without social support network may experience greater burden and psychological distress. For these individuals, neighborhood social cohesion may serve as an alternative protective factor providing an additional layer of social support. Using data from the 2006–2012 Health and Retirement Study (HRS), we examined the association between neighborhood social cohesion and psychological distress among caregiving spouses (N = 1,219), stratified by the availability of a nearby social support network. Approximately 13.5% of caregivers reported having no support network, while 86.5% had children, close friends, or relatives in their neighborhood. Caregivers without a support network exhibited higher levels of hopelessness (M = 2.88, SD = 1.42) and depressive symptoms (M = 3.03, SD = 2.50) compared to those with a support network (hopelessness: M = 2.56, SD = 1.30; depressive symptoms: M = 2.59, SD = 2.30). A series of regression models were estimated, controlling for sociodemographic characteristics, caregiving intensity, and the caregiver-care recipient relationship. Social cohesion was significantly associated with lower psychological distress (hopelessness: b=-0.18, p<.001; depressive symptoms: b=-0.20, p<.01). Notably, the protective effect of social cohesion was stronger among caregivers with no support network (hopelessness: b=-0.20, p<.05; depressive symptoms: b=-0.49, p<.01) compared to those with a support network (hopelessness: b=-0.17, p<.001; depressive symptoms: b=-0.14, p<.05). Findings underscore the importance of fostering social cohesion to mitigate psychological distress, particularly among caregivers with limited social support network. Community-based interventions aimed at strengthening social ties may be beneficial in supporting caregiving spouses.

